# Postoperative Pain and Intravenous Patient-Controlled Analgesia-Related Adverse Effects in Young and Elderly Patients: A Retrospective Analysis of 10,575 Patients

**DOI:** 10.1097/MD.0000000000002008

**Published:** 2015-11-13

**Authors:** Jae Chul Koh, Jinae Lee, So Yeon Kim, Sumin Choi, Dong Woo Han

**Affiliations:** From the Department of Anesthesiology and Pain Medicine, Anesthesia and Pain Research Institute (JCK, SYK, SC); and Biostatistics Collaboration Unit, Yonsei University College of Medicine, Seoul, Republic of Korea (JL).

## Abstract

In this retrospective analysis of 10,575 patients who used fentanyl-based intravenous patient-controlled analgesia (IV-PCA) after surgery, we evaluated difference between young and elderly patients on their characteristic of adverse effects.

We reviewed the data collected from the patients who were provided IV-PCA for pain control following elective surgery under either general or spinal anesthesia between September 2010 and March 2014. Postoperative pain, incidence of PCA-related adverse effects, and risk factors for the need of rescue analgesics and antiemetics for postoperative 48 hours were analyzed.

Pain intensity (numerical rating scale [NRS]) at postoperative 6 to 12 hours (4.68 vs 4.58, *P* < 0.01) and incidence of nausea or vomiting (23.8% vs 20.6%, *P* < 0.001) were higher in young patients, while incidence of PCA discontinuation (9.9% vs 11.5%, *P* < 0.01) and sedation (0.1% vs 0.7%, *P* < 0.001) was higher in elderly patients. Despite larger fentanyl dose used, a greater proportion of young patients required rescue analgesics (53.8% vs 47.9%, *P* < 0.001) while addition of ketorolac was effective in reducing postoperative pain. Despite lower incidence of postoperative nausea and vomiting (PONV), a larger proportion of elderly patients required rescue antiemetics (10.1% vs 12.2%, *P* < 0.001) while addition of ramosetron was effective in reducing PONV.

In conclusion, when fentanyl-based IV-PCA is used for postoperative pain control, a larger proportion of young patients may require rescue analgesics while elderly patients may require more rescue antiemetics. The addition of ketorolac or ramosetron to the PCA of young and elderly patients can be effective to prevent rescue analgesics or antiemetics use.

## INTRODUCTION

Intravenous patient-controlled analgesia (IV-PCA) is one of the most commonly used strategies in modern clinical practice for controlling postoperative pain. It involves continuous administration of a programmed dose of analgesics, while also allowing patients to receive additional, need-based doses. It has played an important role in improving the prognoses of surgery and anesthesia by decreasing postoperative pain and increasing patient satisfaction.^1,2^ However, there have been several reports on its adverse effects, which include inadequate pain control, nausea and vomiting, dizziness, and headache.^3,4^ There have been several studies examining the risk factors for occurrence of PCA-related side effects in an attempt to minimize these adverse effects in susceptible patients.^4–6^

With the increasing number of older surgical patients, it has become more important to understand their patterns of postoperative pain and adverse effects associated with IV-PCA. Elderly patients may differ from young patients in many ways, including physical status, preoperative medication use, and pain intensity.^7^ They may also experience adverse effects different from those experienced by young people after exposure to the same drug or stimulus. In addition, they may have a decreased cardiac output, reduced renal and hepatic function, and increased susceptibility to the central nervous system active medications.^8^ Many studies of IV-PCA have been published since it was introduced for the management of postoperative pain. However, to our best knowledge, there is no prior study about postoperative pain and PCA-related adverse effects comparing elderly and young patients using postoperative IV-PCA. Therefore, in this retrospective study, we investigated and compared the postoperative pain and the incidence of PCA-related adverse effects in young and elderly patients using fentanyl-based IV-PCA, and evaluated the risk factors for the need of rescue analgesics and antiemetics during a 48-hour postoperative period.

## METHODS

This study was approved by the Institutional Review Board (approval no: 3-2015-0070) of Gangnam Severance Hospital, Seoul, Korea and registered at clinical trials. gov (NCT02448862). All the patient's identification information and data were encoded and scrambled in restricted computer to protect privacy of all subjects. The informed consent was waved for this medical record review study, under regulation of the Institutional Review Board.

Since 2010, a PCA service team, consisting of 2 specialized nurses in our hospital, has collected multidisciplinary clinical data from all patients using IV-PCA during a 48-hour postoperative period. They evaluated the clinical outcomes, such as the quality of pain control, the need for additional rescue analgesics or antiemetics, and the presence of any adverse effects, including nausea, vomiting, headache, dizziness, or sedation. Pain intensity scores were measured on a numerical rating scale (NRS: 0–10, with 0 = no pain, and 10 = extreme pain), and the intensity of nausea was graded on an 11-point verbal rating scale (0 = no nausea to 10 = extreme nausea). Rescue drugs were administered according to our institutional guidelines. For rescue analgesics, 30 mg of ketorolac (Keromin; Hana Pharm. Co., Seoul, Korea) or 25 mg of pethidine (Pethidine; Jeil Pharmaceutical Co. Ltd., Daegu, Korea) were administered when pain scores were higher than 4 on the NRS or upon patient request. A rescue antiemetic was provided when the nausea score was higher than 4, when retching or vomiting developed, or upon patient request. A 10 mg dose of metoclopramide (Macperan; Dong Wha Pharm. Co., Ltd., Seoul, Korea) was administered as the first-line rescue antiemetic; patients with persistent and refractory nausea and vomiting were given 4 mg of ondansetron (Onseran; Yuhan, Seoul, Korea) or 0.3 mg of ramosetron (Nasea; Astellas Pharma Korea, Seoul, Korea).

The patients’ demographic variables were analyzed, including age, sex, body mass index, American Society of Anesthesiologists physical status, as well as any history of smoking, motion sickness, postoperative nausea and vomiting, hypertension, and diabetes mellitus. Anesthesia- and surgery-related variables, including duration of anesthesia, type of anesthesia (general or spinal), and laparoscopy and other operation types (categorized as abdominal, thoracic, orthopedic, head and neck, spine, and other) were also analyzed. All the postoperative variables were recorded at 0 to 6 (including stays at the postanesthesia care unit), 6 to 12, 12 to 18, 18 to 24, and 24 to 48 hours postoperatively.

We reviewed the data collected from the patients who were provided IV-PCA for pain control following elective surgery under either general or spinal anesthesia between September 2010 and March 2014. We used a disposable PCA pump (Ambix Anaplus; E-Wha Fresenius Kabi Inc, Gyenoggi-do, Korea, or Accufuser plus; Woo Young Medical, Chungcheong-do, Korea) with 100 mL of fentanyl diluted with saline for the 48-hour PCA infusion. The pump was set at an infusion rate of 2 mL/hour, a bolus dose of 0.5 or 1 mL, and a lockout time of 15 minutes. It was at the anesthesiologist's discretion whether an additional analgesic drug (60–120 mg of ketorolac or 40–160 mg of nefopam), and an antiemetic drug (4–16 mg of ondansetron, 0.3–0.6 mg of ramosetron, or 0.075–0.15 mg of palonosetron) would be added to the PCA, as well as the dosage amount. At the end of the surgery, a 5-hydroxytryptamine receptor 3 antagonist (4 mg of ondansetron) was administered to the patients, and then the IV-PCA was initiated. Patients were divided into young patients group (age 20–39 years) and elderly patients group (age ≥ 70 years). Exclusion criteria were as follows: age less than 20 years or between 40 and 69 years, postoperative ventilator support or intensive care, impaired cognitive function, routine use of analgesic or antiemetic agents due to certain type of surgery or other illnesses, and incomplete data.

All statistical analyzes were performed using SAS 9.1 software (SAS Institute Inc, Cary, NC). We analyzed the demographic characteristics and features of anesthesia and surgery using a *t*-test for continuous variables and a χ^2^ test (or a Fisher exact test) for categorical variables. We performed univariate logistic regression analysis to identify significant predictors of rescue analgesic or antiemetic administration by considering the variables listed above. The factors whose *P* values were less than 0.05 in the univariate logistic model, or were considered clinically important, were included in the multivariate logistic regression analysis. The possibility of multicollinearity between factors under consideration was reduced by excluding the factors whose variance inflation factors were larger than 10. The estimated odds ratios (ORs) with 95% confidence intervals were presented and *P* values less than 0.05 were considered statistically significant.

## RESULTS

**FIGURE 1:**
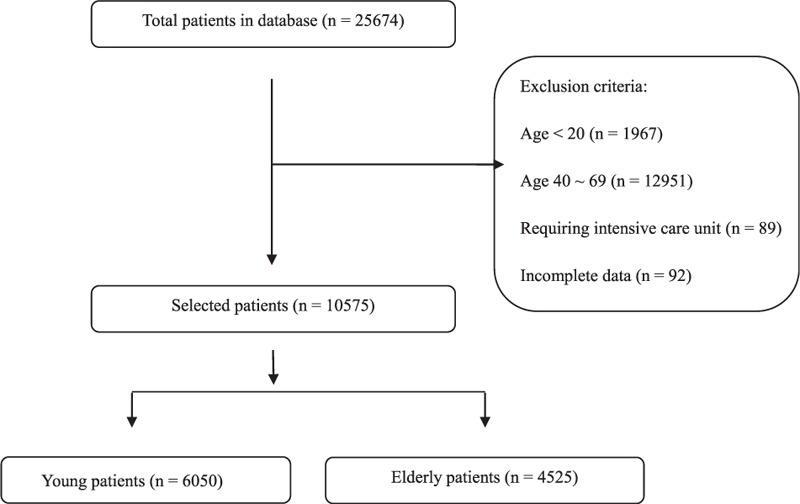
Flow chart indicating both included patients and exclusion criteria.

Among the 25,674 patients for whom data were collected, a total of 10,575 were included in this study (Fig. 1): 6050 in the young patients group (age 20–39 years) and 4525 in the elderly patients group (age ≥70 years).

The flow chart for patient selection is shown in Figure 1. Patients’ demographic characteristics, and the details of PCA, anesthesia, and surgery are described in Table 1. There were more women among young patients than among elderly patients (61.3% vs 47.9%). The number of patients with a history of smoking and an American Society of Anesthesiologists physical status of 3 or 4 were higher in the elderly patients group than in the young patients group. Regarding past history, there was no significant difference between the 2 groups in the incidence of previous postoperative nausea and vomiting (PONV), while motion sickness showed a higher prevalence among young patients compared to elderly patients. Our present analysis showed that background infusion with fentanyl (between 0.11 and 0.93 μg/kg/h in the young patients group, and between 0.05 and 0.87 μg/kg/h in the elderly patients group) had no severe adverse effects in either group. The anesthesiologists had a tendency to use more fentanyl in the IV-PCA for young patients, compared to elderly patients and the mean ± standard deviation (SD) doses of fentanyl used were 17.6 ± 4.0 versus 15.7 ± 4.3 μg/kg, respectively (*P* < 0.001). The proportions of additional analgesics and antiemetics mixed into the PCA were 16.0% versus 11.9% and 88.0% versus 89.5% in young and elderly patients, respectively. The duration of anesthesia was shorter in young patients compared to elderly patients, and the mean ± SD durations were 174.1 ± 110.2versus 213.5 ± 105.6 minutes, respectively (*P* < 0.001). In both groups, general anesthesia was more commonly administered than spinal anesthesia. For maintenance of general anesthesia, an inhalational agent (sevoflurane, desflurane, or isoflurane) was more frequently chosen than propofol in both groups. Nitrous oxide was not used in any case, and remifentanil was infused intraoperatively in most cases of both young (99.1%) and elderly (99.5%) patients. Laparoscopic surgeries were more commonly performed in elderly patients than in young patients (29.48% vs 16.98%, *P* < 0.001), with abdominal surgery the most commonly performed surgery type in both groups.

**TABLE 1:**
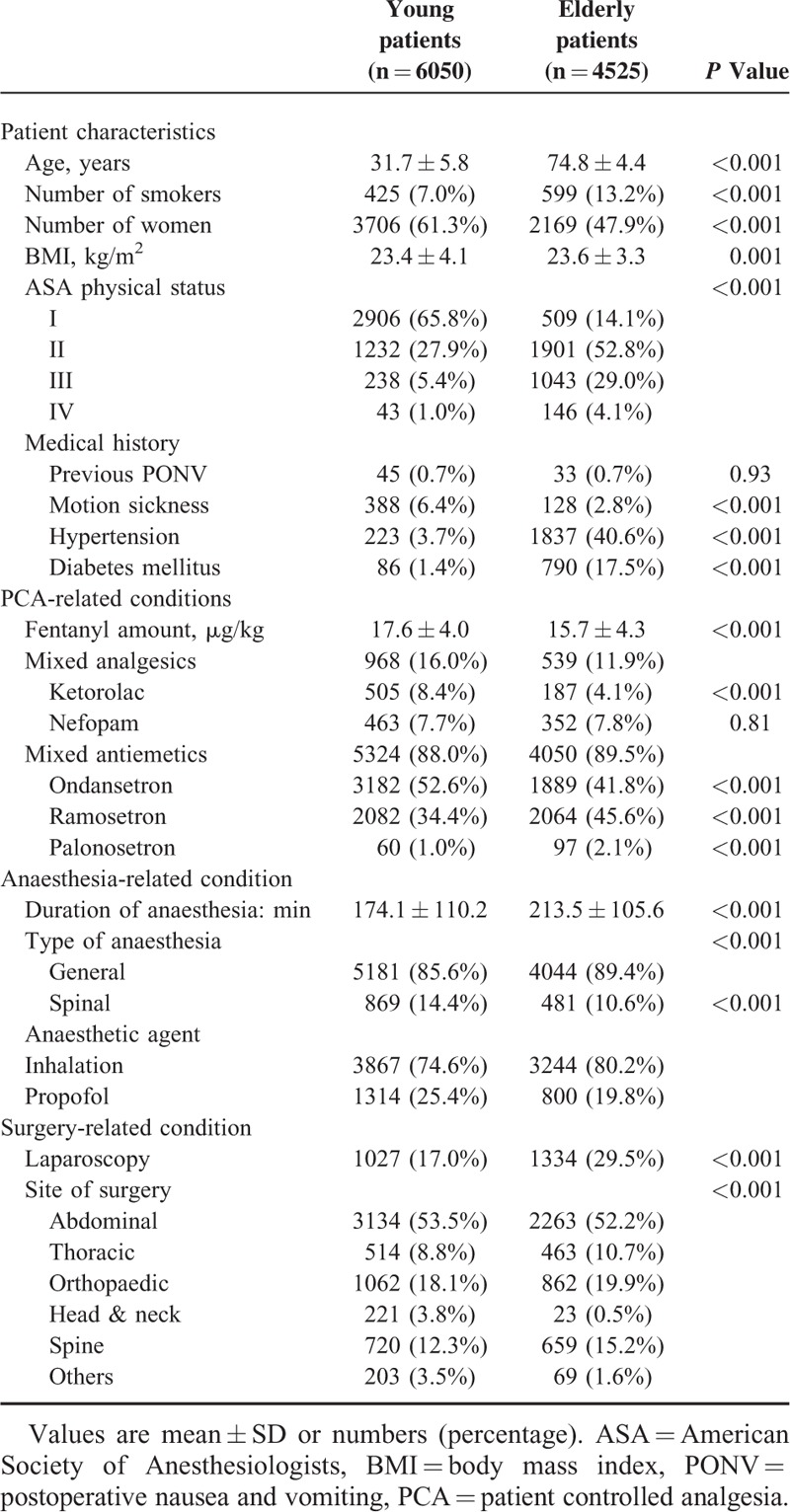
Preoperative Characteristics of Patients Using Fentanyl-Based IV-PCA

The NRS for pain was higher in young patients at 6 to 12 hours postoperatively compared with elderly patients. There was no statistically significant difference at other postoperative time points (Table 2). The proportion of patients who required rescue analgesics at least once during the postoperative 48-hour period was higher in young patients than in elderly patients (53.8% vs 47.9%, *P* < 0.001).

**TABLE 2:**
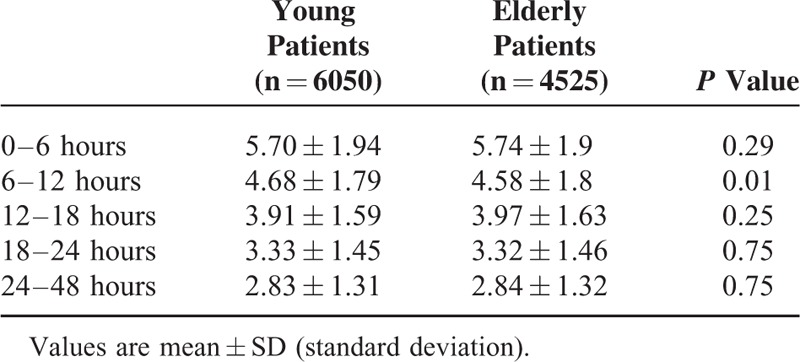
Postoperative Numerical Rating Scale for Pain Intensity

Young patients showed a higher incidence of nausea and vomiting, headache, and dizziness, whereas sedation was more commonly observed in elderly patients (Table 3). However, the proportion of patients who required rescue antiemetics at least once during the postoperative 48-hour period was higher in elderly patients than in young patients (12.2% vs 10.1%, *P* < 0.001). Discontinuation of PCA was more frequently observed in the elderly patients group compared to the young patients group (11.5% vs 9.9%, *P* < 0.01). The causes of PCA discontinuation were mostly nausea (64.6% vs 67.0%) and dizziness (20.6% vs 17.4%) in both young and elderly patients, respectively.

**TABLE 3:**
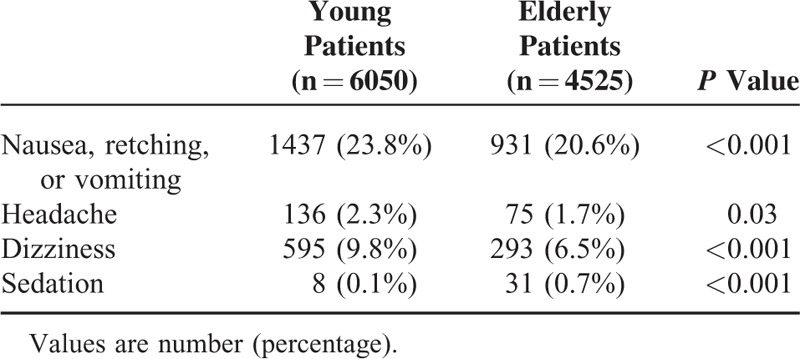
The Incidence of Patient Controlled Analgesia-Related Complications in Young and Elderly Patients

In the multivariate analysis of risk factors for the need of rescue analgesics, female sex was identified as an independent risk factor for an increased need for rescue analgesics, regardless of age group. A history of smoking was identified as an independent factor in young patients, but was not associated with the need for rescue analgesics in elderly patients. The use of ketorolac as an adjuvant mixed in the PCA was identified as an independent factor that could lower rescue analgesic requirements in young patients, but it did not reduce the rescue analgesic requirements in elderly patients. Spinal anesthesia and laparoscopic surgery were not associated with the need for rescue analgesics in either group. When compared with abdominal surgery, thoracic surgery was identified as an independent risk factor for an increased requirement for rescue analgesics in both groups. Orthopedic surgery was also identified as a risk factor associated with the need for rescue analgesics in elderly patients, while head and neck surgery was revealed as a factor for reducing the analgesic requirement in young patients (Table 4).

**TABLE 4:**
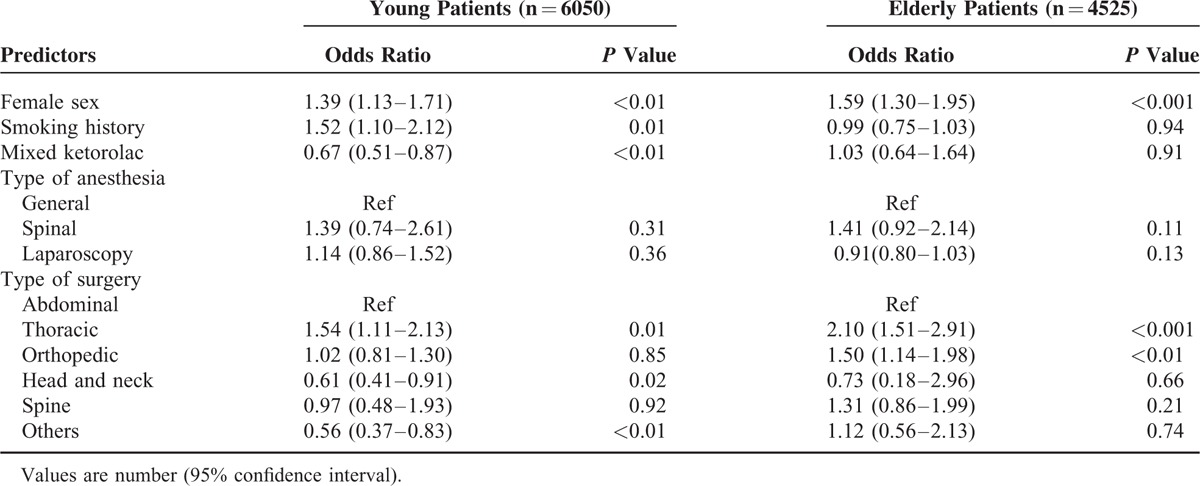
Factors Associated With Rescue Analgesics Requirements

The independent risk factors for the need of rescue antiemetics were also analyzed using multivariate analysis. Female sex was identified as an independent risk factor for increased rescue antiemetic requirement in both groups. The additional use of ramosetron as an antiemetic agent in the PCA was a statistically significant independent factor that could lower the rescue antiemetic requirement in elderly patients but not in young patients. In young patients, spinal anesthesia was an independent factor for reduced antiemetic requirements, whereas laparoscopic surgery was a risk factor for increased rescue antiemetic requirements. Regarding the type of surgery, head and neck surgery was identified as a risk factor for the need of rescue antiemetics in young patients, and orthopedic and spine surgery were identified as risk factors for the need of rescue antiemetics in both groups (Table 5).

**TABLE 5:**
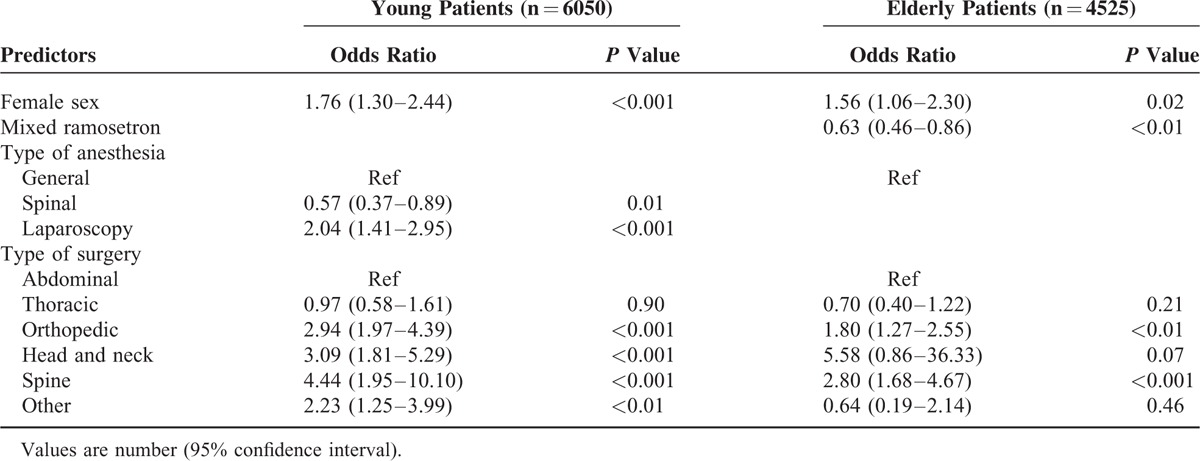
Factors Associated With Rescue Antiemetic Requirement

## DISCUSSION

As elderly patients constitute a large and rapidly increasing proportion of surgical patients, proper management of postoperative pain that minimizes adverse effects is necessary. However, there have been few investigations of the efficacy and adverse effects of postoperative opioid-based IV-PCA in elderly patients. We evaluated and compared the postoperative pain and PCA-related adverse effects in young and elderly patients, and identified the risk factors for the need of rescue analgesics and antiemetics. We found that despite the use of larger doses of fentanyl, a greater proportion of young patients required rescue analgesics than elderly patients and mixing of ketorolac as an adjuvant in PCA was an independent factor that could lower rescue analgesic requirements in young patients. The incidence of the need for rescue antiemetics and discontinuation of PCA was higher in the elderly patients while incidence of nausea or vomiting was higher in young patients. Ramosetron, as an antiemetic agent mixed into the PCA, was also identified as an independent factor that could lower the need for rescue antiemetics.

Fentanyl is well suited for IV-PCA because of its rapid onset time. It is mostly metabolized in the liver into norfentanyl, hydroxy-propionyl-fentanyl, and hydroxypropionyl-norfentanyl (the inactive forms of fentanyl); less than 10% of fentanyl is excreted through the kidneys without being metabolized.^9^ In elderly patients, impaired metabolism of fentanyl due to decreased hepatic blood flow and function can lead to higher plasma concentrations of fentanyl as compared to those in young patients. The brains of elderly patients seem to be more sensitive to fentanyl, as a study by Scott and Stanski^10^ found, which indicated that the amount of fentanyl required to produce the same electroencephalographic stage decreases with increased age. Similar to our findings, most clinicians tend to reduce the dose of fentanyl in IV-PCA for elderly patients on the assumption of age-related differences between young and old patients in the pharmacokinetics and pharmacodynamics of fentanyl. The NRS scores for pain during all postoperative periods were similar between the 2 groups, except that the NRS scores at 6 to 12 hours postoperatively were significantly lower in elderly patients. However, this difference did not seem to be relevant from the clinical point of view. Despite comparable NRSs for postoperative pain, a smaller proportion of elderly patients required rescue analgesics than young patients. One of the possible reasons could be that a high proportion of elderly patients suffer from chronic pain and thus have a tendency to see pain as a normal part of the aging process, although they usually have well-preserved pain sensation.^11^ Several studies have suggested that elderly patients may not seek help, and regard pain as “something that I must live with,”^9,12,13^ thereby reporting lower pain intensities than younger patients.^14,15^

Sex has been considered as an important factor related to pain intensity, that is, higher pain intensity is associated with the female sex. In our study, female sex was identified as an independent risk factor for the need of rescue analgesics in both groups, a finding consistent with previous studies.^16,17^ The mechanism for a relationship between sex and postoperative pain control is multidimensional and includes the influence of neurobiological, psychological, and cognitive factors.^18^

A decrease in the requirement for rescue analgesics was observed when ketorolac was mixed into the IV-PCA in the young patients group. Ketorolac has been used in combination with opioids in multiple studies,^19–21^ as it reduces the opioid requirement dose and its side effects. In the elderly patients in our study, however, the ketorolac added into the PCA did not reduce the requirement for rescue analgesics. This finding might result from the relatively lower dose of ketorolac used in that group compared to the young patients group, as most anesthesiologists would not be willing to administer a high dose of nonsteroidal antiinflammatory drugs to elderly patients, for fear of its adverse effects. The risk and severity of these adverse effects, such as gastric bleeding and renal impairment, are increasing, especially in elderly patients.^22,23^ Nefopam also could be considered as an adjuvant to the analgesics added into IV-PCA, but it did not help reduce the need for rescue analgesics in either group. Although nefopam might reduce postoperative fentanyl consumption with better analgesia, its effect as an adjuvant to opioid analgesia is still controversial, and further investigation with larger dose of nefopam than ours may be needed.^24,25^

Among the type of surgery, thoracic, compared to abdominal, surgery was identified as an independent risk factor for the need of rescue analgesics in both groups. The reason for this result might be the large incision and resection performed during the operation, and hence more aggressive interventions, including additional peripheral nerve blocks or epidural analgesia, could be considered for better management of postoperative pain.

After surgery and anesthesia, nausea or vomiting is often described as a more uncomfortable adverse effect thanpain.^26,27^ The incidence of nausea or vomiting was higher in young patients than in elderly patients, which is consistent with previous findings.^28,29^ According to the report of Belleville et al,^30^ the likelihood of PONV decreases by 13% for each 10-year increase in age. Therefore, the management of PONV in elderly patients may often be overlooked, as they are considered a low-risk group for PONV. However, we found that a larger proportion of elderly patients than young patients required rescue antiemetics. In addition, nausea was the main cause of PCA discontinuation, which was also more common in elderly patients compared to young patients. This finding might be related to the report that found that increased age was an independent factor that increased willingness to pay for PONV treatment.^27^ Considering that improper management of PONV in elderly patients can lead to a failure of IV-PCA as a pain management modality, and elderly patients may tend to be more stoic and less willing to spontaneously express discomfort,^31^ we should not underestimate the occurrence of PONV in elderly patients. According to our results, ramosetron can be considered a more effective antiemetic drug than ondansetron and palonosetron when combined in IV-PCA for elderly patients, although prospective randomized controlled studies will help to confirm our results.

Female sex was also a risk factor for increased nausea or vomiting in both groups, similar to the results of other studies.^4,28,30,32,36,40^ Sex-based differences with respect to the development of PONV may be attributed to variations in serum gonadotropin or other hormone levels, and thus these differences may not be significant in preadolescent age groups or in patients older than 80 years.^32–35^ In our results, the female sex in even elderly patients was an independent risk factor for rescue antiemetic requirement, while its OR (OR: 1.56) was relatively lower than that of young patients (OR: 1.76). It is unclear why elderly female patients still showed a high need for antiemetics, but one possible cause might be that our elderly patients were mostly in their 70s, and thus could potentially still be partially influenced by the hormonal variations associated with the female sex. According to the results of our study, the use of spinal anesthesia was related to decreased requirement for rescue antiemetics in young patients. In addition, the type of surgery could be regarded as a significant predictor of PONV, although that is still debatable. Orthopedic surgery was related to increased requirement of rescue antiemetics in both groups, and laparoscopic surgery, head and neck surgery (including ear, nose, and throat), and dental surgery were risk factors for the need of rescue antiemetics in young patients, similar to previous reports.^28,32,36,37^ However, it was an unexpected finding that spine surgery was a potent risk factor for rescue antiemetics in both groups. One of the possible causes for this finding might be that in our institution, many cases of multilevel spine surgery have been performed that required long surgical times and blood transfusions.

We used a balloon-type PCA device with background infusion; however, its effectiveness and risk factors are still being debated. Although the use of background infusion with morphine is not recommended for elderly patients because of the increased risk of respiratory depression, some studies have found otherwise.^38,39^ In our institution, fentanyl was used in the IV-PCA regimen instead of morphine. This was because it has no active metabolites and displays a wider therapeutic index with fewer opioid-related adverse events than morphine, while there are few reports about background infusion of IV-PCA using fentanyl.^4,5,40^ In our results, respiratory depression was not observed in any patient in either group. For early detection of respiratory depression, monitoring the presence of sedation can be a useful indicator. The overall incidence of sedation in both groups was lower compared to other findings while sedation was more frequently observed in the elderly patients compared to the young patients as expected.^41^ These results suggest that background infusion with fentanyl in the dose range used in our study is relatively safe, without the risk of respiratory depression, even in elderly patients.

This retrospective study has several limitations. In this study, we did not control either the methods or the drugs used during anesthesia, or the type and dosage of adjuvant drugs added to the IV-PCA. However, we think that the results of this study, obtained from multivariate logistic regression, are reliable because of the large sample size in our study. Another limitation is that most of the patients analyzed were either Korean or belonged to another Asian ethnicity, so the results of our study may differ for other races or ethnicities.

In conclusion, when fentanyl-based IV-PCA is used for postoperative pain control, a larger proportion of young patients may require rescue analgesics, despite the use of larger doses of fentanyl compared to those given to elderly patients and the addition of ketorolac to the PCA can be effective for the prevention of rescue analgesic requirement. Elderly patients are more likely to demand rescue antiemetics, despite their relatively lower incidence of PONV compared to young patients and addition of ramosetron as an antiemetic agent into the PCA should be considered in the case of elderly patients, due to its better compliance with a fentanyl-based IV-PCA regimen.
